# A Southern Hemisphere origin for campanulid angiosperms, with traces of the break-up of Gondwana

**DOI:** 10.1186/1471-2148-13-80

**Published:** 2013-04-08

**Authors:** Jeremy M Beaulieu, David C Tank, Michael J Donoghue

**Affiliations:** 1 Department of Ecology and Evolutionary Biology, Yale University, P.O. Box 208106, New Haven, CT 06520-8106, USA; 2 College of Natural Resources & Stillinger Herbarium, University of Idaho, 875 Perimeter Drive MS 1133, Moscow, ID 83844-1133, USA; 3 Institute for Bioinformatics and Evolutionary Studies, University of Idaho, 875 Perimeter Drive MS 3051, Moscow, ID 83844-3051, USA; 4 National Institute for Mathematical and Biological Synthesis, University of Tennessee, 1122 Volunteer Blvd, Ste. 106, Knoxville, TN 37996, USA

**Keywords:** Campanulidae, Campanulids, Biogeography, Gondwana, Southern Hemisphere, Vicariance

## Abstract

**Background:**

New powerful biogeographic methods have focused attention on long-standing hypotheses regarding the influence of the break-up of Gondwana on the biogeography of Southern Hemisphere plant groups. Studies to date have often concluded that these groups are too young to have been influenced by these ancient continental movements. Here we examine a much larger and older angiosperm clade, the Campanulidae, and infer its biogeographic history by combining Bayesian divergence time information with a likelihood-based biogeographic model focused on the Gondwanan landmasses.

**Results:**

Our analyses imply that campanulids likely originated in the middle Albian (~105 Ma), and that a substantial portion of the early evolutionary history of campanulids took place in the Southern Hemisphere, despite their greater species richness in the Northern Hemisphere today. We also discovered several disjunctions that show biogeographic and temporal correspondence with the break-up of Gondwana.

**Conclusions:**

While it is possible to discern traces of the break-up of Gondwana in clades that are old enough, it will generally be difficult to be confident in continental movement as the prime cause of geographic disjunctions. This follows from the need for the geographic disjunction, the inferred biogeographic scenario, and the dating of the lineage splitting events to be consistent with the causal hypothesis.

## Background

With the development of powerful phylogenetic tools for reconstructing geographic range evolution [1-3], there has been renewed interest in the possible impact of past continental movements on modern distribution patterns (Table 1). Much attention has focused on the biogeographic history of the Southern Hemisphere, and the breakup of Gondwana has long been viewed as an important factor underlying the distribution of many different groups across the tree of life (e.g. [4-7]). Plants are no exception; the breakup of Gondwana has long been suggested as a cause of intercontinental disjunctions in Southern Hemisphere plant groups [8].

**Table 1:** Studies that have tested for Gondwanan vicariance in angiosperm groups with Southern Hemisphere distributions

**Clade**	**Taxonomic rank**	**Vicariance supported?**	**Reference**
Magnoliidae			
Atherospermataceae (Laurales)	Family	No	[9]
Winteraceae (Canellales)	Family	Yes/No	[10,11]
Annonaceae (Magnoliales)	Family	Yes/No	[12,13]
Myristicaceae (Magnoliales)	Family	No	[13]
Monocotyledonae			
Burmanniaceae (Dioscoreales*)*	Family	No	[14]
Lilliales (Monocotyledonae)	Order	Yes	[15]
*Ceroxyloideae (*Arecaceae)	Subfamily	No	[16-18]
*Chamaedoreeae* (Arecaceae)	Tribe	No	[19]
*Cocoeae* (Arecaceae)	Tribe	No	[20]
Poales (Commelinidae)	Order	Yes	[21]
Restionaceae (Poales)	Family	No	[22]
*Oreobolus* (Cyperaceae)	Genus	No	[23]
Eudicotyledonae			
Proteaceae (Proteales)	Family	Yes/No	[24-26]
*Gunnera* (Gunneraceae)	Genus	Yes	[27]
*Aristotelia* (Elaeocarpaceae)	Genus	No	[28]
Cunoniaceae (Oxidales)	Family	No	[29]
Malpighiaceae (Malpighiales)	Family	No	[30,31]
Rhamnaceae (Rosales)	Family	No	[32]
Moraceae (Rosales)	Family	No	[33]
*Nothofagus* (Nothofagaceae)	Genus	Yes/No	[34-38]
Myrtaceae (Myrtales)	Family	Yes	[39]
Melastomataceae (Myrtales)	Family	No	[40-42]
Crypteroniaceae (Myrtales)	Family	Yes/No	[43-46]
Burseraceae (Sapindales)	Family	Yes/No	[47,48]
Bombacoideae (Malvaceae)	Subfamily	No	[49]
*Adansonia* (Malvaceae)	Genus	No	[8,50]
Boraginaceae (Lamiidae)	Family	No	[51]
Nepenthes (Nepenthaceae)	Genus	Yes/No	[52,53]
*Exacum* (Gentianaceae)	Genus	No	[54]
*Ourisia* (Plantaginaceae)	Genus	No	[55,56]
*Abrotanella* (Asteraceae)	Genus	No	[57-59]
*Polyscias* (Araliaceae)	Genus	No	[60]

We cite the traditional taxonomic rank of the focal group and whether or not the break-up of Gondwana was supported by the study as the cause of vicariance. Yes/No indicates conflicting results between studies.

As recent studies have examined Southern Hemisphere disjunctions in plants, a common result has been that groups are found to be too young to have been influenced by the Gondwanan breakup (Table 1). Consequently, the view has developed that Southern Hemisphere plant groups exhibiting intercontinental disjunctions may not provide true signatures of Gondwanan vicariance, but rather that these disjunctions have been shaped by more recent events, sometimes involving movements through the Northern Hemisphere (e.g. [30,33,40,61-63]). However, these results for groups that are too young should not be interpreted as ruling out an influence of the breakup of Gondwana. To properly explore this issue we need to focus on clades that extend back into the relevant time frame in the mid- to late-Cretaceous (e.g., [64]). In general, in angiosperms these will be quite large clades, and often more inclusive than the traditional genus and family levels where most previous analyses have focused. Although angiosperm phylogeneticists have worked hard on resolving relationships among the deepest branches (e.g. [65-68]), it is only quite recently that the phylogenies of larger, older, and globally distributed angiosperm clades (e.g., Fabidae, Malvidae, Lamiidae, Campanulidae, *etc.*; *sensu*[69]) are being resolved in sufficient detail to confidently address deep biogeographic problems.

Here we examine the Campanulidae (campanulids), an angiosperm clade of some 35,000 species, and explore the biogeographic history of its major lineages (i.e. Aquifoliales, Asterales, Apiales, and Dipsacales). Despite the great extant species richness of campanulids in the Northern Hemisphere, it appears that many of the earliest diverging campanulid lineages are largely confined to the Southern Hemisphere, suggesting that the group might have initially diversified in the Southern Hemisphere, and might also have been influenced by the break-up of Gondwana. In this study, we combine Bayesian divergence time estimates with a likelihood-based biogeographic method, focusing especially on areas corresponding to the Gondwanan landmasses. As we will show, the reconstructed evolutionary history of the campanulids put them in the right places during the right time periods to have been influenced by Gondwanan events. Overall, we view our analyses as exploring the degree to which convincing conclusions can be reached on early biogeographic events in very large, very old, and very widespread clades.

## Methods

### Taxon and gene sampling

The Campanulidae encompass four major lineages of angiosperms – the Aquifoliales, Asterales, Apiales, and the Dipsacales – as well as a number of smaller clades: Bruniales (Bruniaceae and Columelliaceae), Escalloniaceae *s.l.* (including *Eremosyne*, *Polyosma*, *Tribeles*; Escalloniales *sensu*[70]), and Paracryphiaceae *s.l.* (including *Quintinia* and *Sphenostemon*; Paracryphiales *sensu*[70]). Here, we take advantage of the representative sampling scheme of Tank and Donoghue [71], which included 121 campanulid species – 28 Apiales, 40 Asterales, 30 Dipsacales, 9 Aquifoliales, and 14 species representing the Paracryphiaceae, Escalloniaceae, and Bruniales (*sensu*[71]). To this sample we added 22 outgroups: *Ceratophyllum demersum* and 21 species representing major branches of Eudicotyledonae (eudicots; [69]). Our outgroup sample was selected from the genome-scale chloroplast dataset of Moore et al. [68]. The use of these outgroups allowed the inference of campanulid divergence times in the context of the more inclusive eudicot clade. To minimize the impact of missing data on substitution rate estimates, we chose four chloroplast genes for the dating analysis – three coding regions (*mat*K, *ndh*F, and *rbc*L) and one non-coding region (*trn*L-F). These were selected from the original 10-region cpDNA dataset of Tank and Donoghue [71] and ensured a minimum amount of missing data (<5%).

### Divergence time analysis

Dating analyses were conducted using Markov chain Monte Carlo (MCMC) methods implemented in BEAST (Ver. 1.5.4; [72]). BEAST allows for uncertainty in divergence time estimation by simultaneously incorporating uncertainty in both tree topology and the age of multiple fossil calibrations. Age estimates for fossil calibrations are treated as probabilistic priors, rather than point estimates [73]. An important feature of BEAST is that it employs an uncorrelated relaxed-clock (UCLN) model to estimate divergence times [74]. For each branch, the UCLN independently draws substitution rates from a lognormal distribution, allowing substitution rates to either be autocorrelated (i.e., inherited from parent node to child node) or uncorrelated across the phylogeny, depending on the data.

We assumed a GTR + Γ substitution model applied to the entire data set; we did not partition the data by gene regions. Analyses carried out on the data partitioned by gene never reached convergence and showed symptoms of over-parameterization across several independent runs (i.e., mean rate plummeted to zero after only a few million generations). We supplied a starting tree based on a maximum likelihood analysis of the data using RAxML [75] with branch lengths smoothed to time in r8s [76]. The use of a starting tree overcame the issue of inferring a zero probability during the initial parameter search in BEAST. We ran six independent MCMC runs of 35 million generations, sampling every 1000 generations. To ensure that the posterior distribution of topologies and branch lengths came from the target distribution, convergence and proper sampling of the likelihood surface (effective sample size > 200) of the chains was assessed using Tracer v1.5, with the first 10 million generations discarded as burn-in for each run. Post burn-in samples from the marginal posterior distribution were combined using LogCombiner v1.5.4 [72]. Trees were summarized with TreeAnnotator and the results represent the maximum clade credibility trees with the consensus ages being the median estimate.

### Fossil calibrations

Six fossils were used as minimum-age calibrations, with five providing fossil age estimates for splits occurring within campanulids. Information regarding each fossil, and the minimum age applied, is briefly reviewed below. For all fossils we attached a lognormal prior probability distribution. The lognormal distribution is known to be the most appropriate distribution for describing fossil information [73] because it automatically assigns a higher probability for the true age to be older than the age of the fossil calibration. In practice, however, it is difficult to justify how much older such a distribution should extend from the minimum age. We assessed the sensitivity of our age estimates to different settings of the lognormal distribution. In the first analysis, we set the parameters of the lognormal distribution to ensure that the 95% tail of the distribution encompassed ~15 Myr from the minimum age of each fossil calibration. A wide prior implies that the fossil calibrations strongly underestimate the true ages. Our second analysis assumed that the fossils are a better approximations of the true age by ensuring that 95% of the probability distribution extended only ~5 Myr from the minimum age.

The first fossils attributed to Eudicotyledonae are Late Barremian-Early Aptian (~125.0 Ma) tricolpate pollen grains – a key synapomorphy for the group [77-80]. The first appearance of the tricolpate grains during this time has often been interpreted as signaling the origin of the group and, therefore, has been used as a maximum age constraint (i.e., [66,67,81-84]). However, while these first grains exhibit the same aperture configuration, they also show “considerable structural variety” [80] in exine sculpture. Moreover, the first localities are geographically widespread, with the first occurrences being in several Gondwanan sites (present-day northern and equatorial Africa; [78,79]) and in what is present-day southern England [77]. These observations can be interpreted as supporting the view that the appearance of the tricolpate grains reflects the rise to dominance of the eudicot lineage rather than the origin of the group (cf. [85]). Consequently, they argue in favor of relaxing a hard lower bound of the tricolpate pollen record (also see [86]).

The remaining fossil calibrations mark nodes within the campanulids. As one calibration point we used the first unequivocal macrofossil evidence of the genus *Ilex*, a fossil seed from the earliest Paleogene (ca. 65 Ma) of Central Europe [87]. Owing to the uncertain placement of this fossil within *Ilex* (i.e., whether along the stem or within the crown), we treated this calibration as the first fossil evidence of the Aquifoliaceae (including *Ilex*, *Phyllonoma,* and *Helwingia*). We note that the oldest fossil pollen grains attributed to *Ilex* are from the Turonian of Australia [88], but this report is unpublished and has not been confirmed [89,90].

As another calibration point we used the oldest reliable macrofossil evidence for Araliaceae, a fossilized leaf from the middle Eocene (40.4 Ma) of southeastern North America [91]. This fossil shows an affinity to the modern genus *Dendopanax* based on the lobed leaves, the presence of three accessory cells surrounding the guard cells, and camptodromous secondary venation. Although we did not specifically sample *Dendropanax*, recent phylogenetic studies have suggested that *Dendropanax* is placed somewhere within a clade that contains *Tetrapanax*, *Schefflera*, *Hedera, Tetrasplandra, Pseudopanax,* and *Cussonia*[92]. Based on this information, and our sample of Araliaceae, we treated this fossil as the first fossil evidence for the most inclusive clade that excludes *Aralia* and *Panax*.

The split between *Torricellia* and *Melanophylla* was assigned a minimum bound of 55.8 MA based on fossil endocarps assigned to *Torricellia* from the Paleocene of North America [93]. These endocarps appear to be closely related to modern *Torricellia* based on their spherical shape, smooth surface, and the presence of three endocarp chambers, two of which are quite large [94]. Endocarps of the closely related *Melanophylla* are similar, except that the endocarp surface is not smooth and the shape is more or less elliptical. Despite overall similarities, there are no specific features that link these fossil endocarps to any extant species of *Torricellia*, and we therefore place the fossil along the stem to *Torricellia*.

The oldest fossils assignable to Asteraceae are fossilized flower heads from the Middle Eocene of Patagonia [95]. These fossils are tentatively assigned to the subfamily Mutisioideae (*sensu lato*) based on the morphology of fossilized pollen grains associated with the flowers. The pollen grains of Mutisioideae exhibit a distinctive exine type with an echinate surface and a columellate ecto- and endosexine [96]. Mutisioideae (*sensu lato*), as it is currently defined, is paraphyletic making exact placement of this fossil unclear, though the exine morphology does exclude the possibility of it being placed within the subfamily Barnadesioideae. Therefore, we placed the fossil as occurring along the stem leading to the rest of Asteraceae after the split from Barnadesioideae (represented by *Barnadesia* in our dataset) and we set the minimum age at 47.5 Ma based on the age of the fossil locality [95].

Finally, the split between *Dipelta* and *Kolkwitzia* was constrained to a minimum age of 33.0 Ma. This calibration is based on fossil fruits of *Diplodipelta* spp. from the Eocene of Western North America, which have been placed along the stem of modern *Dipelta*[97]. The expanded bracts subtending the fruits of *Diplodipelta* and *Dipelta* are very similar, but in *Diplodipelta* the fruits are paired as opposed to being solitary in *Dipelta*. Both groups first appear in the Late Eocene, but *Diplodipelta* is absent from the fossil record after the Miocene [97]. Placement of this fossil was based on a presumed sister relationship between *Dipelta* and *Kolkwitzia*[71].

### Reconstructing the biogeographic history of Campanulidae

The biogeographic history of the Campanulidae was estimated under the dispersal-extinction-cladogenesis model (DEC; [3]). The DEC model assumes dispersal-mediated range expansion and extinction-mediated range contraction, with the probability of either event occurring along a particular branch being proportional to the length of that branch and the instantaneous transition rates between geographic areas [2]. We used *lagrange*[3] to obtain the likeliest dispersal scenarios at all internal nodes of the dated consensus tree (with outgroups removed) under the DEC model. In order to assess uncertainty in biogeographic reconstructions due to both topological and temporal uncertainty, we also inferred the likeliest biogeographic scenarios across 1000 randomly chosen trees obtained from the posterior distribution of dated trees (cf. [98]).

We relied on a composite Akaike weight to summarize the biogeographic reconstructions across our posterior distribution of dated trees. This involved modifying *lagrange* to output the likelihood of all possible biogeographic scenarios estimated at a given node, as opposed to only reporting those that are less than two-log likelihood units away from the inferred global likelihood. The composite Akaike weight is the average of the individual Akaike weight for each biogeographic scenario calculated for each tree separately. Thus, we interpret the composite Akaike weight as describing the average relative likelihood of a given biogeographic scenario over a set of alternative scenarios. The evidence of the most favored scenario is judged by examining the evidence ratio for the scenario with the highest Akaike weight versus all other models [99]. The evidence ratio represents the *relative* evidence of one biogeographic scenario being the most supported, which freed us from interpreting the raw composite Akaike weights between competing reconstructions.

We constructed two biogeographic models for Campanulidae. The first involved coding terminals as occurring in the Northern Hemisphere, in the Southern Hemisphere, or in both in order to test whether there was evidence for the campanulids originating in the Southern Hemisphere. The Northern Hemisphere included the Holarctic (including both Palearctic and Nearctic regions) and Southeast Asia. The Southern Hemisphere comprised the historically persistent Gondwanan landmasses (e.g. South America, Australasia, Africa, and Madagascar). The second model delimited six broad areas: sub-Saharan Africa, Madagascar (here including the Seychelles, Reunion, and Mauritius islands), Australasia (including New Guinea, New Caledonia, and Lord Howe island, New Zealand and Tasmania), South America, Southeast Asia, and the Holarctic. New Guinea and New Caledonia were included as part of the Australasian region based on affinities with the flora of Queensland [100]. Likewise, we included New Zealand as part of the Australasian region based on recognized affinities with the flora of Australia [101,102]. There is growing evidence that the New Zealand landmass was mostly submerged during the Oligocene, which would have removed a true Gondwanan signal from the flora [103-106].

In our six area biogeographic model, we did not restrict the number of ranges a lineage can inhabit, and we altered the migration probabilities among geographic areas to reflect changes in connections during the gradual fragmentation of Gondwana. These migration probabilities largely mirror those used by Mao et al. [107] and range from 0.1 for well-separated areas, to 1.0 for contiguous landmasses. We devised separate migration matrices for four discrete time intervals: 105–80 Ma, 80–50 Ma, 50–30 Ma, and 30–0 Ma. The use of non-zero migration probabilities allowed for the possibility that lineages could have a range that include regions that are well separated today but were once connected through Antarctica (e.g., South America-Madagascar). They also allowed for changes in the probability of dispersal between regions that were once separated by large distances before becoming nearly contiguous. For example, some dispersal events observed within campanulid lineages involved movements from Australasia into Southeast Asia and the Holarctic. The close proximity of Australasia and Southeast Asia seen today resulted from a northward migration of the Australian plate during the early Paleogene that began to slow ca. 30 Ma after the Australian plate collided with the Ontong Java plateau [108,109]. Prior to this a large distance separated Australasia and Southeast Asia during the Cretaceous, which we interpret as a potential limitation to the probability of successful dispersals between these two regions. Nevertheless, by setting migration probabilities to be non-zero (i.e., *Pr* = 0.1), we acknowledge the possibility that such a migration was possible during the Cretaceous.

### Widespread taxa

Given our representative sampling of the major campanulid lineages, the presence of highly diverse and often geographically widespread taxonomic groups (e.g., Campanulaceae, Asteraceae, Apiaceae, and Araliaceae) required that we test several alternative geographic coding schemes for the terminals in the six area biogeographic models. For example, the Asteraceae, with some 23,000 species, is represented by only nine “exemplar” species in our dataset. Our first strategy was to code all highly diverse and widespread lineages as occurring in all six areas. We refer to this as the “anything-goes” strategy. This approach might be considered conservative in the sense of not committing to a specific ancestral range for these taxa – the ancestor could have been anywhere. Unfortunately, this has the consequence of potentially lowering certainty in biogeographic inferences throughout the tree. Our second strategy only took into consideration the geographic ranges of the particular genera used in our dataset, ignoring the ranges of any other member of the lineages that they represent. For example, we coded *Helianthus* as being Holarctic based on the current distribution of the genus, despite it being nested well within the subfamily Asterioideae whose extant members occur in all seven regions. We refer to this as the “exemplar” strategy. Our third strategy, and the one that we favor, coded diverse, widespread lineages according to the best current phylogenetic and biogeographic information on the clade being represented. This approach necessarily relies on studies conducted on younger, less inclusive clades in establishing ancestral areas for major campanulid lineages. We call this the “ancestral inference” “strategy” (see “terminals are higher taxa” in Ronquist [110]). For some groups, this strategy was straightforward, as in the case of the Mutisioideae (consisting of ca. 720 spp.) within Asteraceae. Although only one species of *Gerbera* represented this clade in our analyses, previous analyses of the evolution of this group have demonstrated that the early diverging lineages were restricted to South America [111-113]. Therefore, in this case we were coding Mutisoideae, not *Gerbera*, as originating in South America. In other instances, this was not so straightforward, as in the Asteraceae subfamilies Carduoideae (one species representing ca. 2,600 spp), Cichorioideae (three species representing ca. 2,900 spp), and Asterioideae (three species representing ca. 17,000 spp) of the Asteraceae. We coded Carduoideae and Cichorioideae as being Holarctic based on several studies indicating a Mediterranean origin [111,113,114]. However, we coded Asterioideae as occurring in all regions based on the uncertainty of the relationships within (e.g. Senecioneae; [115]) and among the early diverging lineages of the clade (e.g., [111,113]). Our coding of Adoxaceae relied on several previous analyses [116-118], but for *Viburnum* we adopted the recent suggestion of Clement and Donoghue [119] that the group originated in Southeast Asia. In addition to Asteraceae and Adoxaceae, we relied on previous biogeographic hypotheses for Apiaceae (Apiales; [120-123]) and Araliaceae (Apiales; [124]).

## Results

### Phylogenetic and divergence time analyses

The majority rule consensus topology from our BEAST analysis of the combined *mat*K, *ndh*F, *rbc*L, and *trn*L-F dataset was nearly identical to results reported by Tank and Donoghue [71]. This included strong posterior support (PP = 1.0) not only for the backbone relationships, but also for a majority of the relationships among the less-inclusive campanulid lineages. However, we note two minor differences. First, although not strongly supported (PP < 0.50), within Caprifoliaceae *sensu lato* our analysis placed Morinaceae as sister to a combined clade of Linnaeeae-Dipsacaceae-Valerianaceae. Second, within the Escalloniaceae a sister relationship between *Tribeles* and *Polyosma* was only weakly supported (PP = 0.72). These small differences are not surprising given our use of only four of the 10 cpDNA regions analyzed by Tank and Donoghue [71].

The divergence time estimates from the different treatments of the width of the calibration priors yielded generally similar results with respect to the ages of the major nodes within the campanulids. It is noteworthy that the ~15 Myr calibration prior width analyses did result in a 95% HPD surrounding each age estimate that were, in some cases, considerably wider. This was expected, however, as the width of the priors on fossil calibrations are known to influence the width of the posterior distribution of inferred ages, but not necessarily the median of the distribution [125,126]. The effect was most pronounced for the origin of the Eudicotyledonae, with the resulting posterior age distribution seeming unreasonable based on our understanding of the eudicot fossil record, which is considered to be very reliable [80]. When assuming a ~15 Myr prior on this fossil calibration, we cannot reject a mid-Jurassic origin for the eudicots (95% HPD: 127–164 Ma), an age that exceeds the first fossil occurrences from the Late Barremian-Early Aptian (~125 Ma) by nearly 40 Myr. The use of a ~15 Myr prior width also resulted in posterior age distributions that seemed unnecessarily imprecise in their reflection of the uncertainty in the timing of the origin of the major campanulid lineages. Estimates for the origin of the campanulids included ages that also exceeded the first fossil occurrence of eudicots (95% HPD: 95–130 Ma), which given their nested position within the eudicots, seems unlikely.

It also important to consider the influence of the prior width on our biogeographic analysis, particularly when our intention was to evaluate credible cases reflecting the breakup of Gondwana. In our case, increasing the width of the priors would render the conclusion of Gondwanan vicariance more probable by increasing the chance of an overlap with a particular geological disjunction. In fact, we could guarantee a match if we increased the priors enough. In the limit, this would approximate a fossil record so poor that it could not be used to place constraints on the ages of nodes, in which case almost any disjunction between continents could be interpreted as having occurred within the window of Gondwanan events. Although 5 Myr priors may be too narrow when the fossil record is spotty (as with the campanulids), this has the advantage in our case of biasing *against* favoring the break-up of Gondwana as the cause of a particular disjunction. As we are interested here in identifying only the most credible cases, for the remainder of the paper we will focus on ages estimates from analyses where we assume a ~5 Myr prior width on all fossil calibrations. But we acknowledge that in doing so it is possible that we are failing to identify additional disjunctions that would be consistent with the break-up of Gondwana.

**Figure 1:**
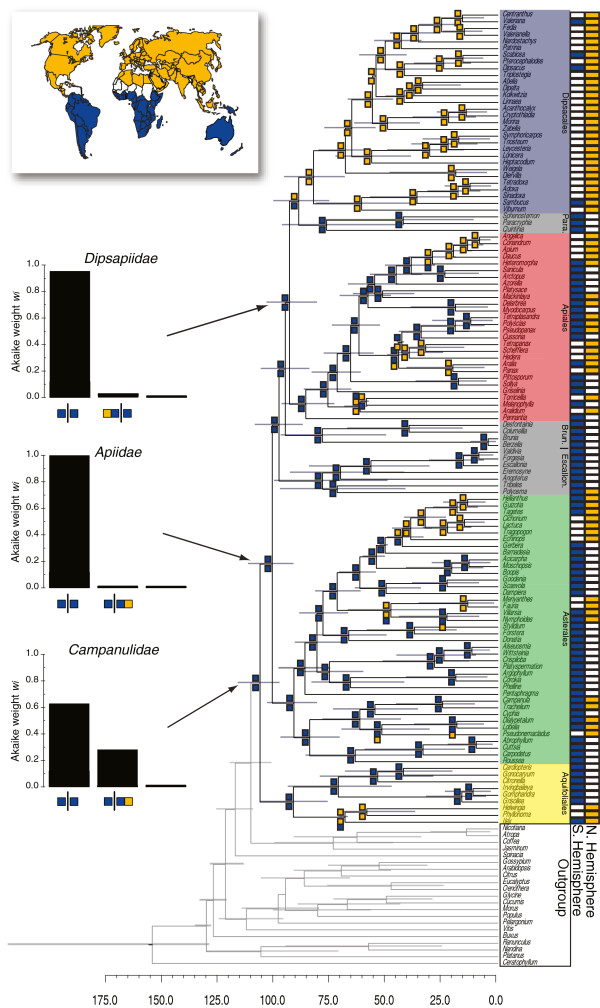
**Divergence time estimates (X-axis, in millions of years) and biogeographic reconstructions for a two-area model for Campanulidae that broadly grouped terminals as occurring in the Northern Hemisphere, the Southern Hemisphere, or both.** The Northern Hemisphere (yellow) included the Holarctic and Southeast Asia. The Southern Hemisphere (dark blue) corresponded to the historically persistent Gondwanan landmasses (e.g. South America, Australasia, Africa, and Madagascar). The ancestral range shown at each internal node (colored boxes) are the reconstructed scenarios with the highest composite Akaike weight were obtained from analyses conducted on 1000 randomly chosen trees from the posterior distribution of dated phylogenies. The three barplots show the three biogeographic scenarios with the highest composite Akaike weight (*w*_*i*_) for the origin of Campanulidae, Apiidae, and Dipsapiidae. Para = Paracyphiaceae; Brun. = Bruniales; Escallon. = Escalloniaceae.

We estimate that the Campanulidae originated in the Albian, centered on 104 Ma (95% HPD: 95–115 Ma; Figure 1). All of the divergences along the campanulid backbone are estimated to have also occurred within the Cenomanian, suggesting rapid succession in the origin of the major campanulid lineages (Figure 1). For example, only a 3 Myr interval is estimated between the origin of the Apiidae (99 Ma; 89–109 Ma; Figure 1) and the next divergence in our tree, the node corresponding to the least inclusive clade containing Bruniales and Dipsacales (96 Ma; 95% HPD: 85–106 Ma). The origin of the Dipsapiidae is the first campanulid divergence with a median age estimate outside of the Cenomanian (91 Ma; 95% HPD: 79–101 Ma; Figure 1).

With the exception of the Dipsacales, the primary campanulid crown clades (i.e. Aquifoliales, Asterales, and Apiales) are inferred to have existed by the end of the Santonian (~83 Ma; Figure 1). The origin of the Asterales was estimated at 89 Ma (95% HPD: 79–101 Ma), though the vast majority of the diversity originated well within the Paleogene, beginning with the emergence of Asteraceae some 49 Ma (95% HPD: 48–52 MA). Likewise, the most diverse clades within Apiales (84 Ma; 95% HPD: 71–95 Ma), Araliaceae (44 Ma; 95% HPD: 41–48 Ma), and Apiaceae (54 Ma; 95% HPD: 43–66 Ma) were estimated to have originated more recently (i.e., early Paleogene).

We estimated the origin of Dipsacales in the early Campanian (80 Ma; 95% HPD: 67–94 Ma). Our median age estimate, though still in the Cretaceous, is younger than previous studies, which suggested an Albian origin [127,128]. However, despite limited sampling, our estimates for several clades nested within the Dipsacales largely agree with more focused analyses (e.g., [98,118,127]). For example, despite sampling only six Valerianaceae species, our median estimate of 42 Ma (95% HPD: 33–54 Ma) is close to the 50 Ma estimate by Moore and Donoghue [118].

In general, our age estimates for campanulids are older than the ages implied by the fossil record (e.g. ~83.5 Ma for crown campanulids; [129]), but younger than estimates from previous molecular studies (e.g. ~123 Ma for crown campanulids; [128]). Previous molecular analyses relied on an autocorrelated model of nucleotide substitution (e.g. [128]), which may have been inappropriate. Indeed, we found no strong evidence for autocorrelated substitution rates (ρ = 0.103; 95% HPD: -0.016 - 0.235) and the standard deviation of the rates was roughly 95% of the mean (coefficient of variation = 0.953; 95% HPD: 0.810 – 1.11). Both of these results are consistent with an uncorrelated model of nucleotide substitution rates.

### Biogeographic reconstructions

Our two-area biogeographic model inferred a campanulid origin in the Southern Hemisphere. A Southern Hemisphere origin was the scenario with the highest composite Akaike weight (*w*_*i*_ = 0.608, evidence ratio = 1.72; Figure 1) relative to all other reconstructed scenarios that did not exceed the confidence window of two-log likelihood units. The second most favored scenario had considerably less weight (*w*_*i*_ = 0.353) and inferred an origin that was widespread across both the Northern and Southern Hemisphere (Figure 1). A Southern Hemisphere origin was also inferred for most of the early campanulid divergences (*w*_*i*_ > 0.90 in all cases), with the one exception being the Dipsacales, which was inferred to have originated in the Northern Hemisphere (*w*_*i*_ = 0.655, evidence ratio = 2.05)*.* In general, movements into the Northern Hemisphere were estimated to have occurred more recently, coinciding with the origin of some of the most diverse clades of campanulids (e.g. Asteraceae, Apiaceae, and Campanulaceae; Figure 1).

When we used the six area biogeographic model, the biogeographic reconstructions were also virtually identical across the three coding strategies for widespread taxa, especially along the backbone of the tree. Thus, the results presented below focus only on our preferred “ancestral inference” coding strategy. We also note that the reconstructions from a model that was unconstrained with respect to changes in connections during the gradual fragmentation of Gondwana were nearly identical to the constrained model, including between-area disjunctions that are consistent with the break-up of Gondwana (see below).

**Figure 2:**
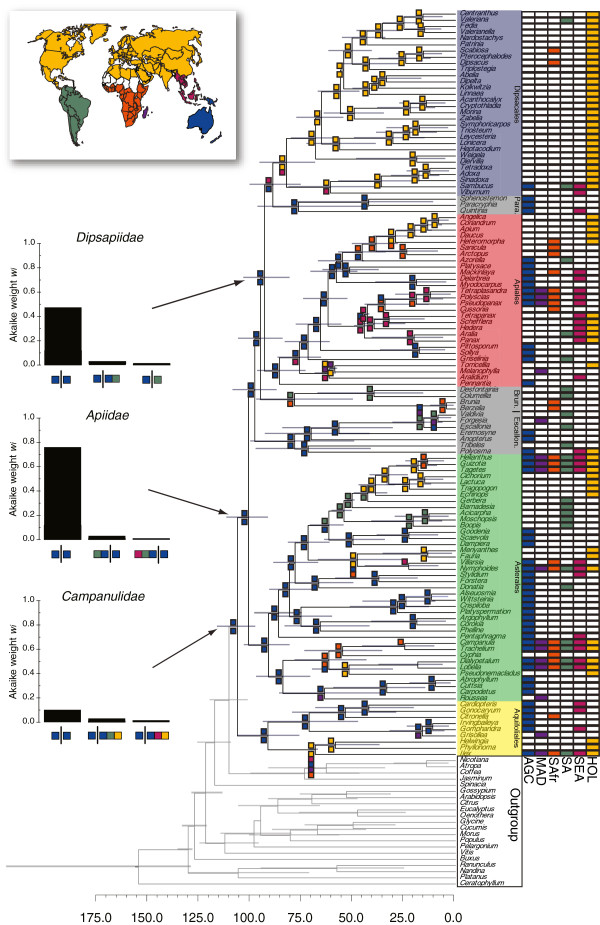
**Divergence time estimates (X-axis, in millions of years) and biogeographic reconstructions for the six-area model for Campanulidae.** The constrained maximum-likelihood biogeographic model included four areas corresponding to Gondwanan landmasses: South America (SA, green), South Africa (SAfr, orange), Madagascar (MAD, purple), and Australasia, including New Guinea, New Caledonia, Tasmania and New Zealand (AGC, dark blue). We included two “northern” regions: Holarctic (HOL, yellow) and Southeast Asia (SEA, magenta). Of the three coding strategies used (see Methods), we depict here the results based on the “ancestral inference” strategy. The ancestral range shown at each internal node (colored boxes) are the reconstructed scenarios with the highest composite Akaike weight obtain analyses conducted on 1000 randomly chosen trees from the posterior distribution of dated phylogenies. The three barplots show the three biogeographic scenarios with the highest composite Akaike weight (*w*_*i*_) for the origin of Campanulidae, Apiidae, and Dipsapiidae. Para = Paracyphiaceae; Brun. = Bruniales; Escallon. = Escalloniaceae.

Our six area biogeographic model estimated that campanulids originated in the Australasian region, containing Australia, New Caledonia, New Guinea, New Zealand, and Tasmania (Figure 2). However, when all alternative scenarios were considered, evidence in favor of the Australasian region was generally low (*w*_*i*_ = 0.05, evidence ratio = 1.84; Figure 2). The second most likely scenario (*w*_*i*_ = 0.03) inferred the origin of the campanulids as being widespread across the Holarctic, South America, and the Australasian regions (Figure 2). There was strong evidence in favor of the initial campanulid divergences taking place in Australasia, implying multiple within-area divergence events prior to movement out of the region (*w*_*i*_ > 0.60; Figure 2).

The first unequivocal movements out of Australasia were reconstructed to have occurred after the origin and early diversification of the primary campanulid clades (Figure 2). The initial divergences within the Aquifoliales were inferred to have occurred within Australasia (e.g. *w*_*i*_ = 0.11, evidence ratio = 2.49 for crown Aquifoliales). In general, there was low support for any one biogeographic scenario for the origin of Aquifoliaceae (*Ilex*, *Helwingia*, and *Phyllonoma*), likely due to the uncertainty created by the widespread distribution of *Ilex* across all six regions. Nevertheless, the scenario with the highest weight (*w*_*i*_ = 0.09, evidence ratio = 1.17) entailed a range expansion to include the Holarctic, Madagascar, South America, and Southeast Asia. This expansion was followed by the *Helwingia-Phyllonoma* clade inheriting the Holarctic portion of this new range, and *Ilex* then dispersing independently into all six regions.

The initial divergences within Asterales were also confidently reconstructed as being within the Australasian region (e.g. *w*_*i*_ = 0.83 for crown Asterales; Figure 2). Previous work has suggested an East Gondwanan origin for the Asterales based on the observation that several small early diverging lineages are, today, largely confined to Australasia [130]. Indeed, a majority of the early divergences within Asterales were associated with Australasia, including branches within Carpodetaceae, Alseuosmiaceae-Phellinaceae-Argophyllaceae, Stylidiaceae, and Goodeniaceae (Figure 2). Several included branches show movement from Australasia into South America (Asteracaceae-Calyceraceae) and into southern Africa (Campanulaceae). Only the branch leading to the Menyanthaceae was reconstructed as being widespread across more than three regions (Australasia, southern Africa, and the Holarctic), though this scenario was not strongly favored (*w*_*i*_ = 0.13, evidence ratio = 1.98). The likeliest scenario for the biogeographic origin of the highly diverse Asteraceae was clearly centered in South America (*w*_*i*_ = 0.84, evidence ratio = 11.1), with nested lineages becoming widespread [111,113,130].

The initial divergence within Apiales was associated with the Australasian region (*w*_*i*_ = 0.86, evidence ratio = 17.1; Figure 2). Subsequent divergences leading to the bulk of Apiales diversity (e.g. Pittosporaceae, Araliaceae, and Apiaceae) also took place in Australasia. However, there is no biogeographic scenario for the origin of Araliaceae that is strongly supported over all others, although most scenarios involve a widespread distribution across Southeast Asia and several Southern Hemisphere regions, including Australasia. Subsequent divergences within nested lineages of Araliaceae are inferred to have taken place primarily within Southeast Asia (Figure 2). The only clade within the Apiaceae inferred to be predominantly Holarctic is the Apioideae. The Holarctic distribution followed a divergence event that severed an ancestral range that included both sub-Saharan Africa and the Holarctic (*w*_*i*_ = 0.69, evidence ratio = 4.69).

The likeliest scenario for the biogeographic origin of Dipsacales was inferred to be within both the Holarctic and Southeast Asia (*w*_*i*_ = 0.57, evidence ratio = 3.78). The movement of Dipsacales into these Northern Hemisphere regions was facilitated by a range expansion of the Dipsidae clade (the least inclusive clade containing Paracryphiaceae and Dipsacales [71]) into Southeast Asia from Australasia (Figure 2). Interestingly, the timing of the movement of the Dipsidae clade (87 Ma; 95% HPD: 74–98 MA) into Southeast Asia predates any direct connection between Australasia and Southeast Asia by nearly 50 Myr. However, we infer such a range expansion into Southeast Asia from Australasia as being somewhat supported over alternative scenarios showing movement of the Dipsidae clade into the North Hemisphere through some other route (i.e., Australasia - > South America - > Holarctic). The Adoxaceae was inferred to be widespread across the Holarctic and Southeast Asia (*w*_*i*_ = 0.56, evidence ratio = 5.81), owing especially to our coding of the origination of *Viburnum* within Southeast Asia [119]. The remaining lineages within the Dipsacales were strictly associated with the Holarctic region (Figure 2).

### Vicariance events associated with the break-up of Gondwana

Given that the origin of campanulids extends back into the Cretaceous and that early diverging campanulid lineages were present in the Southern Hemisphere (Figures 1 and 2), we specifically assessed whether any of the between-area disjunctions were consistent with the break-up of Gondwana.

Although rifting between east and west Gondwana began in the Jurassic, connectivities among areas likely persisted for some time [131]. The first separation began when the India-Madagascar-Seychelles block began to rift from the Australia-Antarctica block during the early Cretaceous (130-120 [131,132]). The Kerguelen Plateau appears to have maintained a connection between the India-Madagascar-Seychelles block and the southern continents, especially Australia-Antarctica, until the beginning of the Late Cretaceous (~100-90 Ma 74-76). Based on our biogeographic reconstruction, the likeliest scenario (*w*_*i*_ = 0.76) for the branch leading to *Roussea*-Carpodetaceae (Asterales) involved a split between Madagascar and Australia, with *Roussea* originating in Madagascar and the rest of Carpodetaceae inheriting the northern Australasian portion of the range (Figure 2). However, because both the median age estimate (63 Ma) and its associated variance (95% HPD: 35-84 Ma) occur well after the presumptive separation of these landmasses, a Gondwanan vicariance explanation for this event seems highly unlikely.

**Figure 3:**
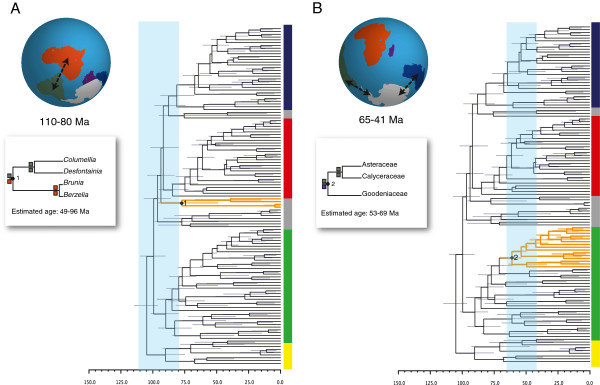
**Possible vicariance events (orange branches) showing both temporal and biogeographic congruence with the breakup of Gondwana.** (**A**) Within Bruniales, the divergence between South American Columelliaceae (*Columellia*, *Desfontainia*) and South African Bruniaceae (*Brunia*, *Berzelia*) overlaps the timeframe (light blue rectangle) for the separation of Africa (orange) and South America (green). (**B**) The divergence between the Australasian Goodeniaceae and the originally South American Calyceraceae + Asteraceae overlap the timeframe (light blue rectangle) for the separation of South America (green) and Australasia (dark blue) from Antarctica during the Early Paleogene.

By contrast, a likely biogeographic scenario (*w*_*i*_ = 0.42) inferred for the first divergence within the newly recognized Bruniales [71] suggests an association with the separation of Africa from South America (Figure 3). First, the disjunction pattern itself suggests such vicariance, with Bruniaceae (represented here by *Brunia* and *Berzelia*) in southern Africa and Columelliaceae (*Columellia* and *Desfontainia*) in South America (northward into Central America). Second, such a vicariance explanation is feasible from the standpoint of our age estimates. Considerable debate remains regarding the sequence leading to the separation between Africa and South America [133]. Three general models have been proposed: (1) the “Samafrica” model, which suggests that South America and Africa (comprising west Gondwana) separated from east Gondwana during the Early Cretaceous (~120 Ma; [133,134]); (2) the “Africa-first” model, which suggests that Africa separated from Gondwana independent of South America [131,135]; and (3) the “Pan-Gondwana” model, which posits that major passages connecting all major Gondwana landmasses, including a trans-Atlantic passage between Africa and South America, were severed more or less simultaneously at the end of the early Cretaceous (~90 Ma; [6]). Despite their differences, all of these models suggest that the physical separation between Africa and South America occurred sometime during the end of the Early Cretaceous or earliest Late Cretaceous (~110-80 Ma; Figure 3). Our median age estimate for the divergence between Bruniaceae and Columelliaceae is 77 Ma, with a variance from 49–98 Ma (95% HPD). Because the upper age estimate fall within the 110–80 Ma range assumed for the separation of Africa and South America (Figure 3), we cannot reject the hypothesis that the disjunction in Bruniales resulted from the rifting of Africa from South America.

The establishment of the Drake’s Passage during the Oligocene (~41 Ma; [136]) completed the separation of the remaining Gondwanan landmass that consisted of Australia, Antarctica, and South America. However, this separation had begun by the beginning of the early Eocene, when the South Tasman Rise became submerged such that a small seaway separated Australia from Antarctica (~55-50 Ma). The separation of Australia and Antarctica became progressively pronounced throughout the Eocene as Tasmania cleared East Antarctica [137,138]. The separation of South America from the Antarctic Peninsula near the end of the Eocene and the early Oligocene (~41 Ma; [136]) marked the opening of the Drake’s Passage and allowed the establishment of the Antarctic Circumpolar Current [131,138]. This was quickly followed by extensive glaciation of Antarctica, though some hypothesize that the development of the Antarctic ice sheets occurred prior to the opening of Drake’s Passage [138,139]. Given this overall scenario, any direct connections between South American and Australia would have been severed between 55–41 Ma, if not earlier.

There is one clear disjunction in the campanulids involving South America and Australasia, the divergence between Goodeniaceae and the combined Calyceraceae-Asteraceae clade. In the most favored biogeographic scenario (*w*_*i*_ = 0.88, evidence ratio = 13.9), Goodeniaceae remained in Australasia, while the Calyceraceae-Asteraceae inherited the South American portion of the widespread ancestral range (Figure 3). We estimate the median age of this split to be 60 Ma (95% HPD: 53–69 Ma). In as much as this age range overlaps the 65–41 Ma range for the separation of Australia from South America, we cannot reject the hypothesis that the split between the Goodeniaceae and Calyceraceae-Asteraceae was promoted by the physical separation of these landmasses (Figure 3).

## Discussion

Our analysis of the biogeography of the Campanulidae indicates that the early evolution of this major angiosperm clade took place in the Southern Hemisphere, perhaps initially in Australasia. Specifically, the Aquifoliales, Asterales, Bruniales, Escalloniaceae, and Apiales all appear to have originated in the Southern Hemisphere, despite the fact that many of these groups are today most diverse in the Northern Hemisphere. It is difficult to infer the ancestral environment in which campanulids initially diversified. However, it is possible that they occupied, at an early stage, more seasonal and temperate climates, especially in view of the high paleo-latitudes of Gondwana at that time [140] and presumed seasonal fluctuations in temperature and photoperiod. Adaptation to such climates may be reflected in a possible synapomorphy of the campanulid clade [69], namely xylem vessel elements with predominately scalariform perforation plates, which are today most commonly found in cold climates [141].

There remains, however, the possibility that the campanulids originated in the Northern Hemisphere or, perhaps, were even widespread across both the Northern and Southern Hemispheres. A Northern Hemisphere origin would argue for the Southern Hemisphere as a center of survival of early-diverging lineages rather than a center of origin. Perhaps the early northern representatives of basal campanulid lineages all went extinct, possibly because the environments that they occupied became too rare. And, perhaps the representatives of these lineages that moved into the Southern Hemisphere survived there because these environments remained intact. Such a scenario would gain support if there were ancient fossils of these lineages in the North Hemisphere. In general, our inferred southern origin for particular clades is consistent with current evidence from the fossil record. For example, there are fossils that not only place Asteraceae in South America in the early Paleogene, but also suggest that they were derived from ancestors that were also Gondwanan in origin (Figure 2; [95,142,143]). However, there is one case that could imply an early northern origin. The Late Cretaceous flowers of *Silvianthemum suecicum* and *Bertilanthus scanicus* would place campanulids in Sweden in the Late Santonian-Early Campanian (~85 Ma; [144]). These fossils share several curious features with modern day *Quintinia* (Paracryphiaceae), such as secretory hairs on the floral surface, styles with radiating palisade cells, the postgenital union of the 3–4 apocarpous styles, and parietal placentation [145,146]. There are, however, several other important features that do not fit, such as stamens opposite the petals, dorsifixed anthers, and, in the case of the *Sylvianthemum*, having 10 stamens in two whorls. These characters raise the possibility that these fossils are not directly related to *Quintinia*, but belong elsewhere within Asteridae (e.g., within Cornales). In fact, our biogeographic analyses might be viewed as supporting such a conclusion. Extant *Quintinia* are today largely restricted to Australasia and our analysis never identified a scenario for Paracryphiaceae involving the Holarctic. At the present time there is no definitive fossil evidence placing any of the early campanulid lineages in the Southern Hemisphere during the Cretaceous, but neither is there conclusive fossil evidence placing them in the Northern Hemisphere. In the absence of such fossil evidence, a southern origin for the campanulids appears to be the best-supported hypothesis.

A corollary of a southern origin hypothesis is that there have been movements within the campanulids from the Southern Hemisphere into the Northern Hemisphere. At this stage we can estimate only a minimum number of such movements; a better understanding of the real number will require more extensive sampling and biogeographic analyses within each of the major lineages. Likewise, identifying pathways of movement from the Southern Hemisphere to the Northern Hemisphere will require fine-grained analyses. If our speculation is correct that the initial diversification of campanulids occurred in more temperate climates of the Southern Hemisphere, it is possible that some early lineages were preadapted to occupy temperate zones in the Northern Hemisphere, and that they tracked such habitats northward when relevant connections allowed successful movement into the Northern Hemisphere (cf. [147]). In any case, northward movements would have been facilitated during the Paleogene by several corridors, such as the connection of South America to North America through the paleo-Caribbean (as postulated for Asteraceae: [111,113]), and the collision of the Arabian Peninsula with Eurasia during the late Eocene (~35 Ma; [148]), which corresponds, for example, to an inferred northward movement within Apiaceae (median = 28 Ma, 95% HPD: 18–40 Ma).

The movement of the Dipsidae (the least inclusive clade containing Paracryphiaceae and Dipsacales; [71]) into the Northern Hemisphere during the Late Cretaceous is perhaps the most difficult to explain. We infer that the range of the ancestor of the Dipsacales and the Paracryphiaceae came to encompass the Austral region and the Southeast Asia between 98 and 73 Ma when there was quite certainly *not* a direct connection between these areas. A closer connection between Australasia and Southeast Asia was established only much later, in the Paleogene [108,109]. It is noteworthy that a Southeast Asian pathway is consistent with recent evidence on basal relationships and biogeographic patterns in Adoxaceae, where there are now indications of the existence of early-diverging Southeast Asian lineages in both *Sambucus*[118] and *Viburnum*[119]. Recent evidence in *Viburnum*, in particular, points to a possible Southeast Asian origin and several movements from there into temperate Asia [119]. If our biogeographic scenarios and age estimates were correct, the establishment of a distribution including the Austral region and Southeast Asia in the Cretaceous would most likely have entailed long distance dispersal. However, the inference of such an event prior to any direct connection could also be pointing to an instance where our age estimates, at least for the origin of the Dipsacales, are simply too old.

Importantly, our dating analyses indicate that the campanulids, and the major lineages within the clade, existed far enough back into the Cretaceous for their diversification to have been influenced by the break-up of Gondwana. As might be expected, therefore, we have been able to identify several disjunctions within early diverging campanulid lineages (e.g. Bruniales and the split between Goodeniaceae and Asteraceae + Calyceraceae; Figure 3) that are consistent with the hypothesis that the separation of the Gondwanan landmasses played a causal role. By “consistent with” we specifically mean that the geographic disjunction, the inferred biogeographic scenario, and the dating of the lineage splitting events are all consistent with the vicariance hypothesis. With respect to the dating, in particular, the inferred range of dates for the lineage splitting event must overlap the range of dates assigned to the relevant geological event based on other evidence. Of course, the need to be consistent in all three ways does highlight the difficulties of pattern-based approaches such as the one adopted here. Although this would appear to be the most stringent test, it is possible that some apparently positive cases will be better explained in the end by dispersal when, for example, taxon sampling is improved. At the same time, data from other sources (e.g., on dispersal limitations) could further bolster a positive conclusion. In any case, the approach we have taken here provides a baseline for more detailed analyses of individual clades.

Viewed from this perspective, it is clear why it is difficult to confidently attribute clade diversification to ancient continental movements. First, plant groups that are old enough to have been influenced by these events are also quite likely to have moved around a lot since that time, becoming widespread across several biogeographic regions (e.g. *Ilex*; Figure 2). Such movements will tend to cover up the initial geographic disjunction and, therefore, confound the inference of ancestral areas. We simply will not be able to recognize many genuine cases of ancient vicariance by virtue of subsequent geographic movements. Along these lines, dispersal limitations in some lineages (possibly Bruniales?) may allow a Gondwanan signal to persist and thus bias in favor of detecting such disjunctions. Second, as we move that far back in time, confidence intervals on age estimates tend to become wider. On the one hand, this will increase the chance of false overlap with the geological event in question, potentially yielding false positive results. In fact, this will be especially true for clades where the quality of the fossil record is poor. This will increase confidence intervals further and make almost any disjunction between continents appear to have occurred within the window of a particular geological event. On the other hand, as the range of possible lineage splitting dates widens, many additional geological and climatological events might be responsible, so that causal factors become harder to identify with confidence. This can be especially problematic when there is the added uncertainty associated with the age of the continental disjunctions themselves, as is the case with the breakup of Gondwana [133]. In view of these potentially confounding issues, in general, it is remarkable that we have uncovered even a single possible case of vicariance.

## Conclusions

One general implication of this work concerns the way in which biogeographic studies have been pursued. Studies are typically carried out on traditional taxonomic groups, at traditional taxonomic ranks (genera, families, *etc.*), which happen to exhibit a particular disjunction pattern, usually without any initial regard for the age of the chosen group in relation to the presumed age of the causal event of interest. Accordingly, it is not too surprising that studies in angiosperms have often refuted continental drift as an explanation (Table 1). As it emerges, most of the genera and many of the families of angiosperms are simply not old enough to have been significantly influenced by the movement of continents. On the whole, these end up being cases of pseudo-congruence, and this has fostered the growing belief that continental movements have had little to do with angiosperm disjunctions and diversification, and that either dispersion through the Northern Hemisphere or long-distance dispersal must be the main explanations (e.g., [61]). However, the paucity of examples of Gondwanan vicariance in plants may largely reflect our rank-oriented focus on groups that end up not being old enough to be relevant for this particular biogeographic question.

The alternative approach is to take whatever age information is available directly into account, even at the stage of selecting study groups to address particular biogeographic problems (cf. [149]). That is, if we are interested in understanding the influence of ancient geological events, we need to choose lineages that extend back into the relevant time periods. If we are interested in exploring the possible influence of the break-up of Gondwana, it makes sense to select large and potentially ancient group, such as campanulid angiosperms. Not surprisingly, the best examples of southern ancestry and the impact of Gondwana involve larger, older clades, such as birds [4], mammals [5], and several other large angiosperm groups (e.g., [21,150]).

A natural corollary is that in conducting global biogeographic analyses it may often be necessary to move beyond traditionally recognized genera and families, and gravitate instead to much larger, more inclusive clades, most of which will not have been formally named. In this respect it is noteworthy that the several possible examples of Gondwanan vicariance that we have highlighted here all involve lineages that have only recently been discovered and are either unnamed (e.g., the split between Goodeniaceae and Asteraceae + Calyceraceae) or only recently named (e.g., Bruniales). If we continue to focus attention on off-the-shelf taxonomic groups at particular Linnaean ranks to address biogeographic problems, we may continue to overlook the best evidence for the impacts of ancient geological events on the genesis of biodiversity.

## Competing interests

The authors declare that they have no competing interests.

## Authors’ contributions

JMB, DCT, MJD contributed to the conceptualization and design of the study, contributed to the collection of data, and contributed to analyses and writing of the manuscript. All authors read and approved the final manuscript.
